# Uridine phosphorylase 1 is a novel immune‐related target and predicts worse survival in brain glioma

**DOI:** 10.1002/cam4.3251

**Published:** 2020-06-24

**Authors:** Jin Wang, Shihai Xu, Wen Lv, Fei Shi, Shanshan Mei, Aijun Shan, Jianzhong Xu, Ying Yang

**Affiliations:** ^1^ Department of Emergency Shenzhen People's Hospital (The Second Clinical Medical College, Jinan University; The First Affiliated Hospital, Southern University of Science and Technology) Shenzhen People's Republic of China; ^2^ Department of Pediatrics Futian Women and Children Health Institute Shenzhen People's Republic of China

**Keywords:** glioma, immune response, prognosis, UPP1

## Abstract

Uridine phosphorylase 1 (UPP1) has been reported as an oncogene in several malignancies. In glioma, the role of UPP1 remains unclear. This study was performed to explore its role in glioma at transcriptional level. Totally, 998 glioma patients with clinical data were enrolled, including 301 mRNA microarray data from Chinese Glioma Genome Atlas (CGGA) dataset and 697 RNAseq data from The Cancer Genome Atlas (TCGA) dataset. Statistical analysis was performed with R language. UPP1 expression level was positively correlated with WHO grade of glioma. UPP1 was significantly upregulated in mesenchymal subtype and could serve as a potential biomarker for this subtype. Based on most correlated genes of UPP1, Gene ontology analysis revealed that UPP1 was profoundly associated with immune and inflammatory response. Gene Sets Variation Analysis was further performed and showed that UPP1 was particularly correlated with MHC‐II and LCK, which were mainly associated with activities of antigen‐presenting cells and T cells. Moreover, UPP1 was found to be synergistic with various immune checkpoint members, especially with PD1 pathway and B7‐H3. Finally, Kaplan‐Meier curves revealed that higher UPP1 indicated significantly shorter survival for glioma patients. Taken together, UPP1 played an oncogenic role in glioma via suppressing tumor‐related immune response.

## Graphical abstract

UPP1 is positively associated with WHO grade of glioma. UPP1 is mainly involved in tumor‐induced immune response, particularly associated with antigen‐presenting cell‐related immune response and T‐cell activation. High expression level of UPP1 predicts poor prognosis in glioma.

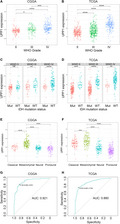

## 1 INTRODUCTION

In brain, glioma is the most common primary tumor in adult patients.^1^ Despite improvements in diagnosis and treatment, the prognosis for glioma is still unfavorable, especially in glioblastoma (GBM).^2^, ^3^ In recent years, lots of work has been done to explore the interaction between glioma tumorigenesis and immune microenvironment.

Uridine phosphorylase 1 (UPP1) has been reported as an oncogene across a range of malignant tumors, including colorectal cancer (CRC),^4^ esophageal squamous cell carcinoma (ESCC),^5^ thyroid carcinoma,^6^ pancreatic cancer,^7^ oral squamous cell carcinoma,^8^ and breast cancer.^9^ UPP1 is an enzyme that catalyzes the reversible phosphorylation of uridine (or 2'‐ deoxyuridine) to uracil and ribose‐1‐phosphate (or deoxyribose‐1‐phosphate). The encoded enzyme functions in the degradation and salvage of pyrimidine ribonucleosides. Recently, UPP1 is reported to play a vital role in immune and inflammatory biological process during particular events such as chronic atrophic gastritis^10^ and respiratory allergy.^11^ Several studies have explored the relationship between the expression level of UPP1 and prognosis in tumor patients. The results concordantly revealed that higher UPP1 level in tumors was usually accompanied with a poorer prognosis and a shorter survival.^5^, ^6^, ^8^, ^9^

However, the role of UPP1 in glioma remains unclear. Thus, we enrolled and analyzed glioma samples from Chinese Glioma Genome Atlas (CGGA) dataset and The Cancer Genome Atlas (TCGA) dataset, aiming at characterizing UPP1 expression in glioma molecularly and clinically.

## 2 MATERIALS AND METHODS

### 2.1 Sample and data collection

Transcriptome data and clinical data of glioma patients were available in CGGA website (http://www.cgga.org.cn/) and TCGA website (http://cancergenome.nih.gov/). In total, 998 glioma samples, including 301 CGGA microarray data and 697 TCGA RNAseq data, were enrolled into this analysis. The baseline characteristics of patients in both cohorts were described in Table S1.

### 2.2 Statistical analysis

Before analysis, RSEM normalized RNAseq data from TCGA were log2 transformed. CGGA microarray data (normalized using GeneSpring GX11.0 as described in CGGA website) were directly analyzed. Statistical analysis was performed with R language. Several R packages including pROC, pheatmap, corrgram, circlize, and survival were used to generating figures. All statistical tests were two‐sided and *P* value <.05 was considered to be statistically significant.

## 3 RESULTS

### 3.1 UPP1 was increased in GBM and decreased in isocitrate dehydrogenase (IDH) mutant glioma

**FIGURE 1:**
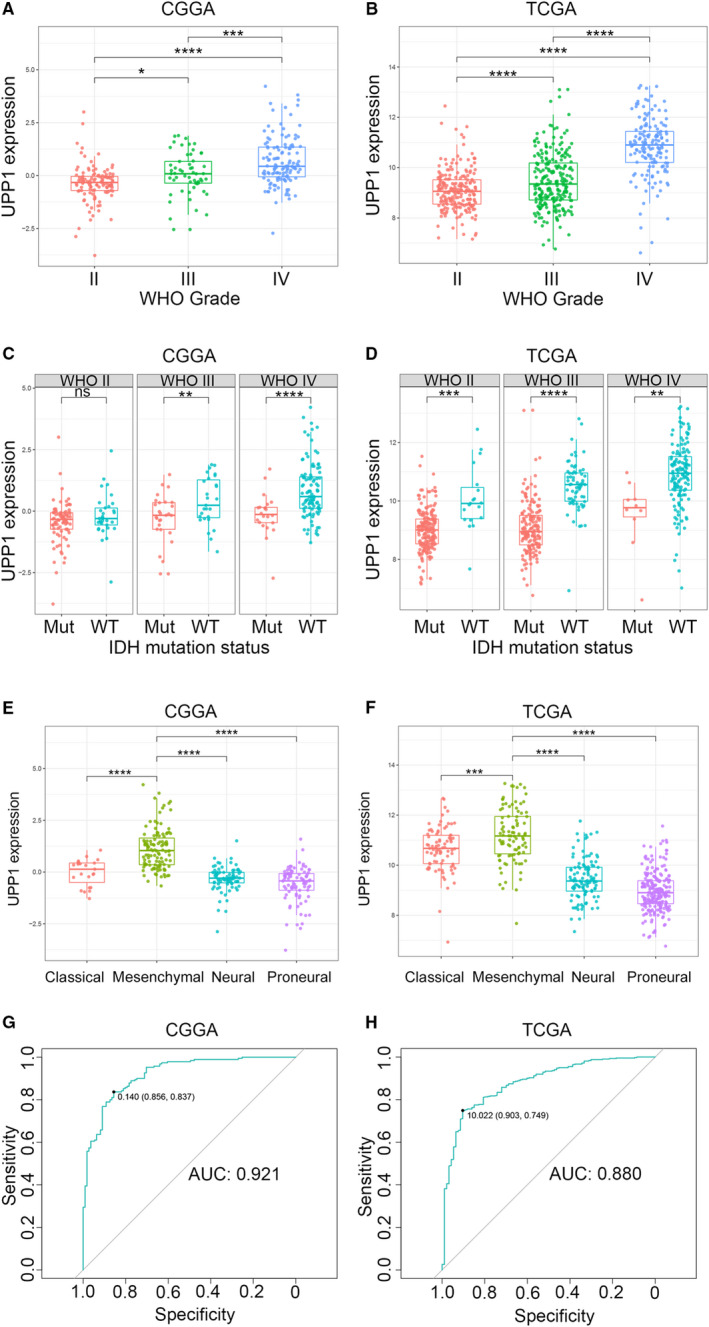
UPP1 expression in CGGA and TCGA dataset according to WHO grade (A, B), IDH mutation status (C, D), TCGA molecular subtype (E, F), and ROC curves (G, H) for predicting mesenchymal subtype. *P value <.05, **P value <.01, ***P value <.001, ****P value <.0001

Uridine phosphorylase 1 expression levels were compared across different WHO grades. In both CGGA and TCGA dataset, the expression level of UPP1 showed a significantly positive correlation with WHO grade of glioma (Figure 1A,B), suggesting that higher UPP1 level was paralleled with higher malignancy in glioma. In addition, when comparing the two groups defined by IDH mutation status, UPP1 expression was found to be significantly upregulated in IDH wildtype glioma than that in IDH mutant type across different WHO grades in both datasets, except for WHO grade I in CGGA (Figure 1C,D). This indicated that UPP1‐related biological process were more involved in IDH wildtype, representing a more aggressive and malignant type of glioma.

### 3.2 UPP1 was upregulated in mesenchymal subtype

Uridine phosphorylase 1 expression levels were compared among different TCGA molecular subtypes. Mesenchymal subtype, the most aggressive type of glioma, showed universally higher expression level of UPP1 than that of other less malignant subtypes in both datasets (Figure 1E,F). Subsequent ROC analyses revealed that UPP1 may function as an effective biomarker for identification of mesenchymal subtype glioma (Figure 1G,H), with the AUC of 92.1% in CGGA and 88.0% in TCGA.

### 3.3 UPP1‐related biological process

**FIGURE 2:**
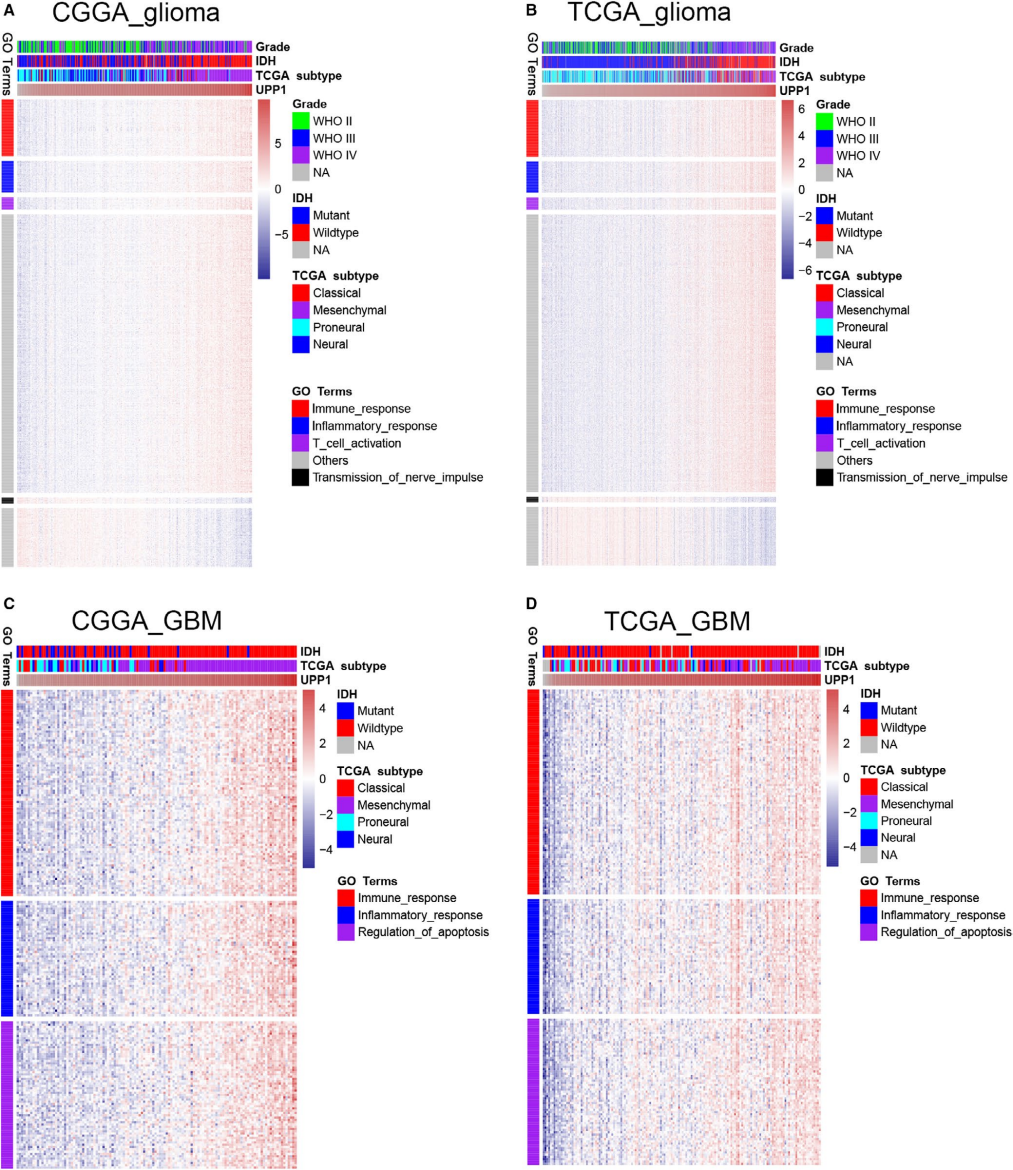
Gene ontology analysis for UPP1 in whole WHO grade glioma (A, B) and GBM (C, D). CGGA, Chinese Glioma Genome Atlas; TCGA, The Cancer Genome Atlas; IDH, isocitrate dehydrogenase; GBM, glioblastoma; UPP1, uridine phosphorylase 1

To explore the biological features of UPP1 in glioma, Pearson correlation test was performed between UPP1 and every single gene. With the criteria of Pearson |*r*| > .5, 2227 genes in CGGA and 2137 genes in TCGA were selected as significantly related genes of UPP1. We chose genes that were shared by both datasets from these significantly related genes and then put them into Gene ontology (GO) analysis (DAVID, https://david.ncifcrf.gov/). We found that UPP1‐positively correlated genes were mainly involved in immune and inflammatory response, especially in T‐cell activation (Figure 2A,B). While UPP1‐negatively correlated genes were more associated with normal biological process, such as transmission of nerve impulse. These results indicated that UPP1 was induced as an immune suppressor in glioma in which tumor‐related immune and inflammatory response were relatively activated.

Uridine phosphorylase 1‐related biological processes were further evaluated in GBM. We found that, besides inflammatory and immune response, UPP1 also showed robust correlation with regulation of apoptosis (Figure 2C,D), which reflected the involvement of UPP1 in anti‐apoptosis nature in GBM.

### 3.4 UPP1‐related immune and inflammatory activities

**FIGURE 3:**
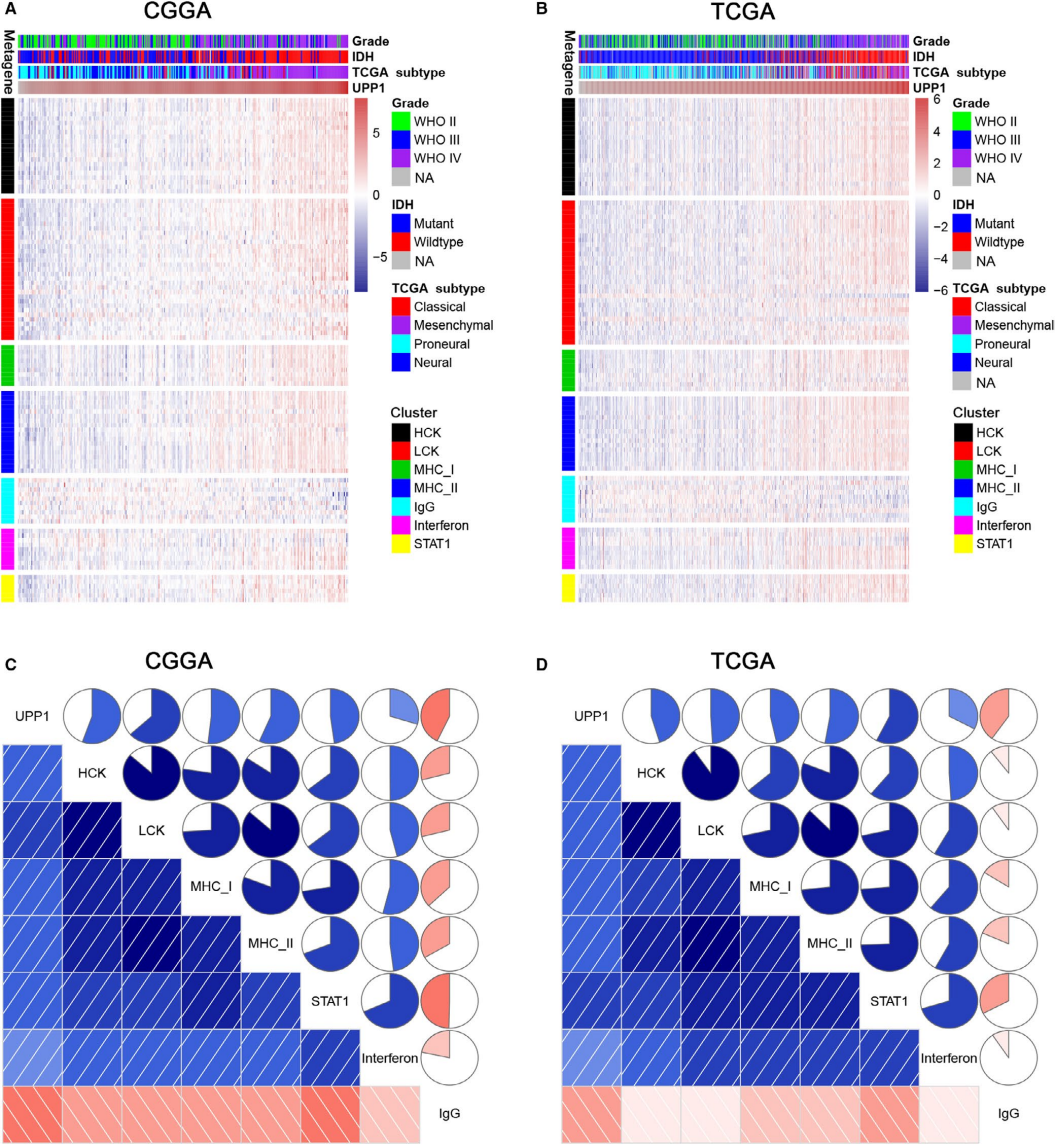
Clusters (A, B) and gene sets variation analysis (C, D) of UPP1‐related immune response. CGGA, Chinese Glioma Genome Atlas; IDH, isocitrate dehydrogenase; TCGA, The Cancer Genome Atlas; UPP1, uridine phosphorylase 1

To further investigate UPP1‐related immune and inflammatory activities, seven clusters including 104 genes, representing different types of immune and inflammatory activities, were defined as metagenes^12^ (Table S2) and subsequently put into Gene Sets Variation Analysis (GSVA).^13^ As shown in Figure 3A,B, we observed that UPP1 expression was positively correlated with most clusters, except for IgG, which was specific for B‐cell immune activity. Corrgram plots derived from GSVA results demonstrated that UPP1 was positively correlated with HCK, LCK, MHC‐I, MHC‐II, interferon, and STAT1, especially with LCK and MHC‐II (Figure 3C,D), which represented specific immune activities of T cells and antigen‐presenting cells (APC), respectively.

### 3.5 UPP1 interacted with immune checkpoint members in glioma

**FIGURE 4:**
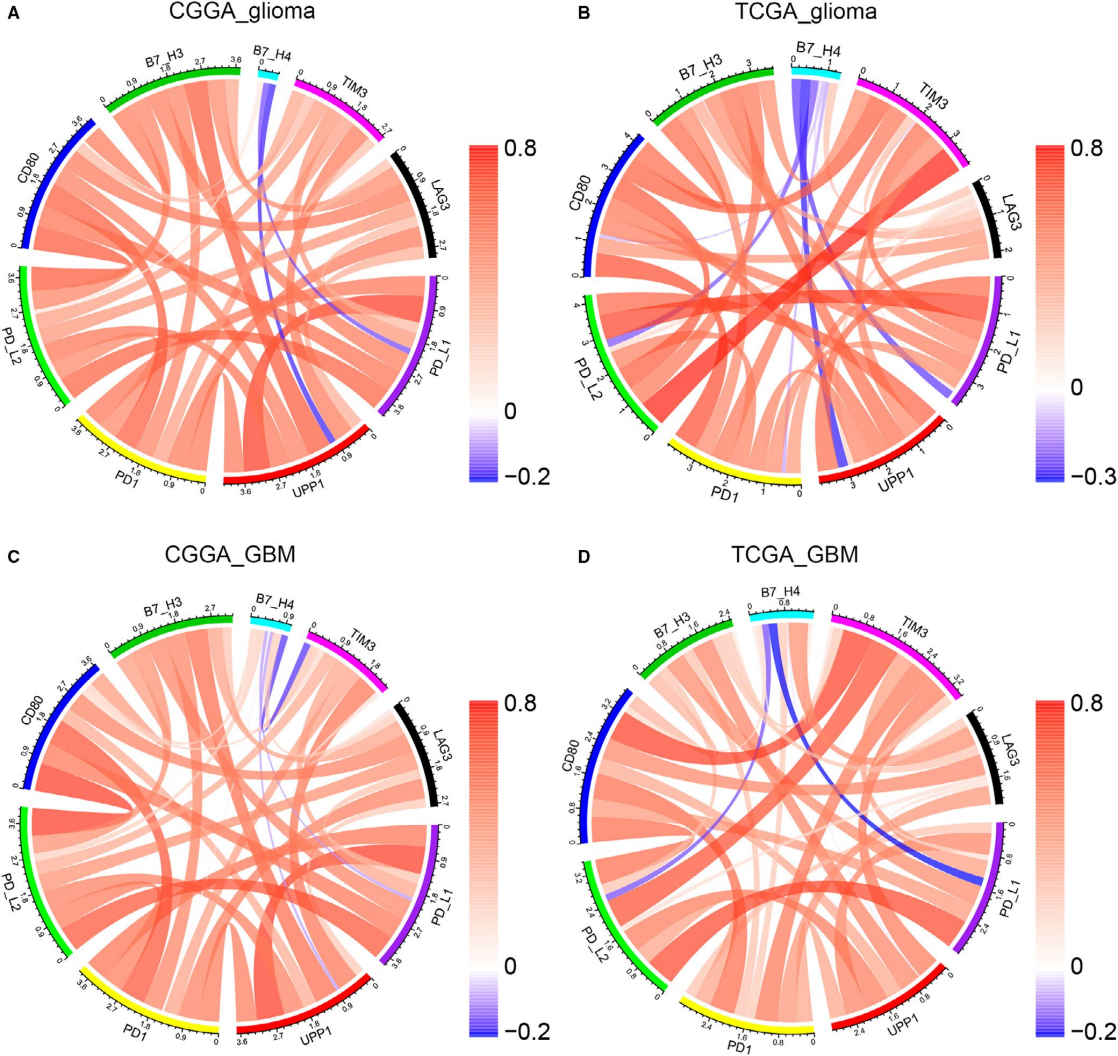
Correlation between UPP1 and immune checkpoint members in whole grade glioma (A, B) and GBM (C, D). CGGA, Chinese Glioma Genome Atlas; GBM, glioblastoma; TCGA, The Cancer Genome Atlas; UPP1, uridine phosphorylase 1

To further investigate the relationship between UPP1 and immune checkpoints, we put UPP1 into Pearson correlation test together with checkpoint members including B7‐H3, B7‐H4, PD1, PD‐L1, PD‐L2, CD80, LAG3, and TIM‐3. Circos plots based on Pearson *r* value demonstrated that the interaction between UPP1 and immune checkpoint members was robust, exhibiting synergistic effects of these key markers during immune response of glioma (Figure 4A‐D).

### 3.6 UPP1 predicts shorter survival for glioma

**FIGURE 5:**
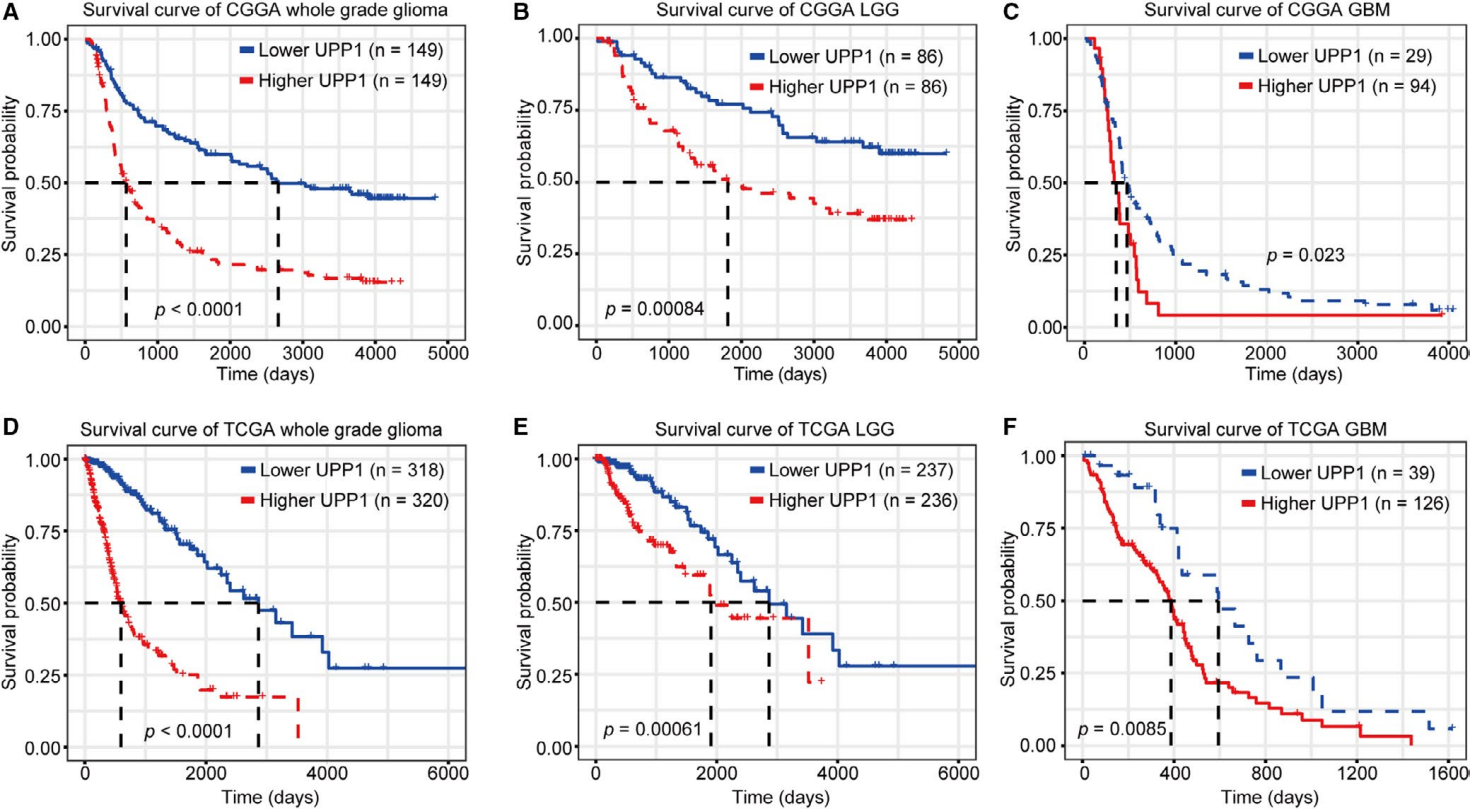
Survival analysis for UPP1 in whole grade glioma (A, D), LGG (B, E), and GBM (C, F). CGGA, Chinese Glioma Genome Atlas; GBM, glioblastoma; TCGA, The Cancer Genome Atlas; LGG, lower grade glioma; UPP1, uridine phosphorylase 1

The prognostic value of UPP1 for glioma was investigated based on Kaplan‐Meier survival analysis. According to median UPP1 expression, glioma patients were divided into two groups in each dataset. As shown in Figure 5A,D, patients who had overexpression of UPP1 in tumors had a significantly shorter survival time than those with lower UPP1 expression. A similar pattern was observed in the Kaplan‐Meier curves of lower grade glioma (LGG) (Figure 5B,E) and GBM patients (Figure 5C,F). This suggested that UPP1 may serve as a negative prognosticator in glioma.

## 4 DISCUSSION

Glioma is the most common brain tumor in adult patients. Despite improvements in treatment including surgery, radiotherapy and chemotherapy, patients still only have a very limited survival time after diagnosis.^14^, ^15^ In the past decade, immunotherapy has been proposed as a novel method for treating malignancies, bringing new hopes for tumor patients, but with inconsistent responding rate across different tumors.^16^ The microenvironment of central nervous system is relatively immune‐privileged and immunosuppressive. Due to such a special microenvironment, tumor‐induced immune response shows distinct pattern between glioma and other malignancies.^17^ Thus, identification of key immunity‐related molecules in glioma is of great necessity.

Uridine phosphorylase 1 expression has been reported to be associated with several malignant tumors, including CRC,^4^ ESCC,^5^ thyroid carcinoma,^6^ pancreatic cancer,^7^ oral squamous cell carcinoma,^8^ and breast cancer.^9^ To address the role of UPP1 in glioma, we carried out this bioinformatic analysis based on the two large cohorts of glioma samples from CGGA and TCGA project. Totally, 998 glioma samples were included into our analysis. UPP1 expression was found to be significantly increased in higher WHO grade, IDH wild type, and mesenchymal subtype, suggesting that UPP1 was positively correlated with much more malignant and aggressive biological process. In addition, ROC curves showed that UPP1 could contribute to discriminate mesenchymal subtype as a potential biomarker.

Gene ontology analysis revealed that UPP1 was predominantly associated with immune and inflammatory response, especially in T‐cell activation. Subsequent GSVA analysis with seven immunity‐related gene clusters suggested that UPP1 expression was positively associated with MHC‐II and LCK, which were specific with activities of APCs and T cells, respectively. Immune response in glioma is induced by tumor‐related biological process, especially in such a relatively immune‐privileged microenvironment of central nervous system. As tumor occurs and grows, T cells and APCs are activated to suppress tumor growth and development. Based on the results reported above, we therefore speculate that upregulation of UPP1 expression inhibits T‐cell activation and APCs‐related immune response, leads to the formation of immunosuppressive microenvironment, and finally, contributes to immune evasion of tumor. However, further study is needed to elucidate the function of UPP1 in the tumorigenesis of glioma.

Heretofore, several important immune checkpoint members were supposed to play a key role in tumor‐related immune response, such as PD1, PD‐L1, PD‐L2, CD80, B7‐H3, B7‐H4, TIM‐3, and LAG3.^18^ Our results revealed that UPP1 interacted synergistically with these checkpoint members, especially positively correlated with PD1 pathway and B7‐H3, which were also associated with suppressive effect on T‐cell activities in tumors. Furthermore, in GBM, the most aggressive type, UPP1 participated in the regulation of apoptosis, corresponding to active anti‐apoptosis characterization of GBM, which further validated the oncogenic effect of UPP1 in glioma.

Survival analysis showed that patients with higher UPP1 expression level lived a significantly shorter survival than the counterparts. This result further validated that UPP1 inhibited immune response, facilitated to formation of an immunosuppressive microenvironment, contributed to the progression of tumor growth, and led to a poor prognosis for glioma patients.

In conclusion, UPP1 was significantly upregulated in glioma, especially in glioblastoma, and exhibited considerable predictive value for prognosis. UPP1 was mainly involved in immune and inflammatory response. In this bioinformatics analysis, UPP1 was identified as a novel molecule that was supposed to play a vital role in glioma. However, a limitation of this study was that no biological validation was performed. Further experimental and clinical studies are warranted for elucidating its role in glioma.

## CONFLICT OF INTEREST

The authors declare that they have no conflict of interests.

## AUTHOR CONTRIBUTIONS

Ying Yang, Jin Wang, Aijun Shan, and Fei Shi made substantial contributions to the study conception and design. Jin Wang, Jiangzhong Xu, and Shanshan Mei participated in data acquisition. Jin Wang, Shihai Xu, and Wen Lv performed data analysis, drafted the manuscript and revised it critically. All authors have read and approved the final manuscript.

## Supporting information

Table S1

Table S2

## Data Availability

The data used to support the findings of this study are available from the corresponding author upon request.
